# Prolonged presence of viral nucleic acid in clinically recovered COVID-19 patients was not associated with effective infectiousness

**DOI:** 10.1080/22221751.2020.1827983

**Published:** 2020-10-27

**Authors:** Ke Hong, Wei Cao, Zhengyin Liu, Ling Lin, Xing Zhou, Yan Zeng, Yuan Wei, Li Chen, Xiaosheng Liu, Yang Han, Lianguo Ruan, Taisheng Li

**Affiliations:** a Department of Infectious Diseases, Jin Yin-tan Hospital, Wuhan, People’s Republic of China; b Department of Infectious Diseases, Peking Union Medical College Hospital, Chinese Academy of Medical Sciences, Beijing, People’s Republic of China

**Keywords:** COVID-19, SARS-CoV-2, prolonged viral RNA presence, infectiousness, secondary transmission

## Abstract

Prolonged presence of viral nucleic acid was reported in certain patients with coronavirus disease 2019 (COVID-19), with unclear clinical and epidemiological significance. We here described the clinical and epidemiological characteristics of 37 recovered COVID-19 patients with prolonged presence of viral RNA in Wuhan, China. For those who had been discharged and re-admitted, their close contacts outside the hospital were traced and evaluated. The median age of the 37 patients was 62 years (IQR 50, 68), and 24 (64.9%) were men. They had common or severe COVID-19. With prolonged positive RT-PCR, most patients were clinically stable, 29 (78.4%) denied any symptoms. A total of 431 PCR tests were carried out, with each patient at a median of 8 time points. The median time of PCR positivity to April 18 was 78 days (IQR 67.7, 84.5), and the longest 120 days. 22 of 37 patients had been discharged at a median of 44 days (IQR 22.3, 50) from disease onset, and 9 had lived with their families without personal protections for a total of 258 person-days and no secondary infection was identified through epidemiological investigation, nucleic acid and antibody screening. Infectiousness in COVID-19 patients with prolonged presence of viral nucleic acid should not solely be evaluated by RT–PCR. Those patients who have clinically recovered and whose disease course has exceeded four weeks were associated with very limited infectiousness. Reconsideration of disease control in such patients is needed.

## Introduction

The pandemic of coronavirus disease 2019 (COVID-19) has continued to grow globally since its outbreak last year. Caused by a novel coronavirus SARS-CoV-2, COVID-19 has exhibited distinctive features from prior coronavirus related diseases. SARS-CoV-2 has demonstrated the capacity to replicate actively in the upper respiratory tissue even before the onset of clinical symptoms, with the first week post infection exhibiting the highest level of viral shedding from the respiratory tract [1,2]. Although most infected patients had reached viral RNA conversion in the upper respiratory samples within three weeks of infection [2,3], the longest duration of respiratory viral shedding from infected individuals and their associated contagiousness remain unclear.

There have been reports that clinically improved patients with negative SARS-CoV-2 RNA testing were later incidentally tested positive. In one study including 70 patients with COVID-19, 21% clinically recovered patients with two consecutive negative results of nucleic acid detection experienced a later positive testing for SARS-CoV-2, and the longest duration of viral RNA positivity in this study was 45 days following infection [4]. This discordance was interpreted as a result of false negative of RT-PCR test and prolonged nucleic acid conversion in these patients. There were also identified patients with consistent positive viral RNA results [5]. Some factors have been associated with prolonged viral RNA positivity, including male sex, delayed admission to hospital after illness onset, and invasive mechanical ventilation [6]. However, little was known regarding the clinical and epidemiological significance of these patients. In most circumstances, infectiousness of patients with prolonged viral positivity but recovered symptoms is still a situation worth serious consideration, and strict quarantine measures are required.

Chronicity of acute respiratory viral infection including prior coronaviruses was rarely reported. Theoretically, positive RT-PCR results could only indicate the presence of selected SARS-CoV-2 nucleic acid sequences in testing samples. It is not clear if such presence indicated disease persistence or relapse in these patients, or whether these patients still possess the capacity of viral transmission. Answers to these questions are critical for the strategy development in public measures for COVID-19 prevention. To clarify the virological and epidemiological features of these patients, we described the virological features in a group of patients who have been followed since the diagnosis of COVID-19 in early the pandemic and have remained positive in viral RNA testing. Information from our study will provide more perspectives for management of clinically recovered patients with prolonged respiratory SARS-CoV-2 RNA.

## Methods

### Study design and participants

Since the outbreak of COVID-19 in China from late 2019, Jin-Yin tan Hospital has been a designated medical centre for treating patients with COVID-19 in Wuhan, Hubei province, China. A total of 2860 COVID-19 patients were hospitalized and followed in this hospital since the epidemic, and those with persistent or intermittent viral RNA positivity in respiratory samples (including the nasopharyngeal, oropharyngeal and sputum samples) for at least 4 weeks were included in our study, regardless of the age and their clinical status. A total of 37 patients were recruited, and all were confirmed SARS-CoV-2 infection diagnosed from 16 January to 29 February 2020.

For all patients, the date of disease onset was defined as the day when the symptoms were noticed, and the date of diagnosis was defined as the day when nasopharyngeal or oropharyngeal swabs were tested positive for SARS-CoV-2. Upon diagnosis and admission, all patients were treated according to the Chinese Recommendations for Diagnosis and Treatment of Novel Coronavirus (SARS-CoV-2) Infection (Pilot 4th version) [7]. The last follow-up of all patients was on April 18.

**Figure 1.**
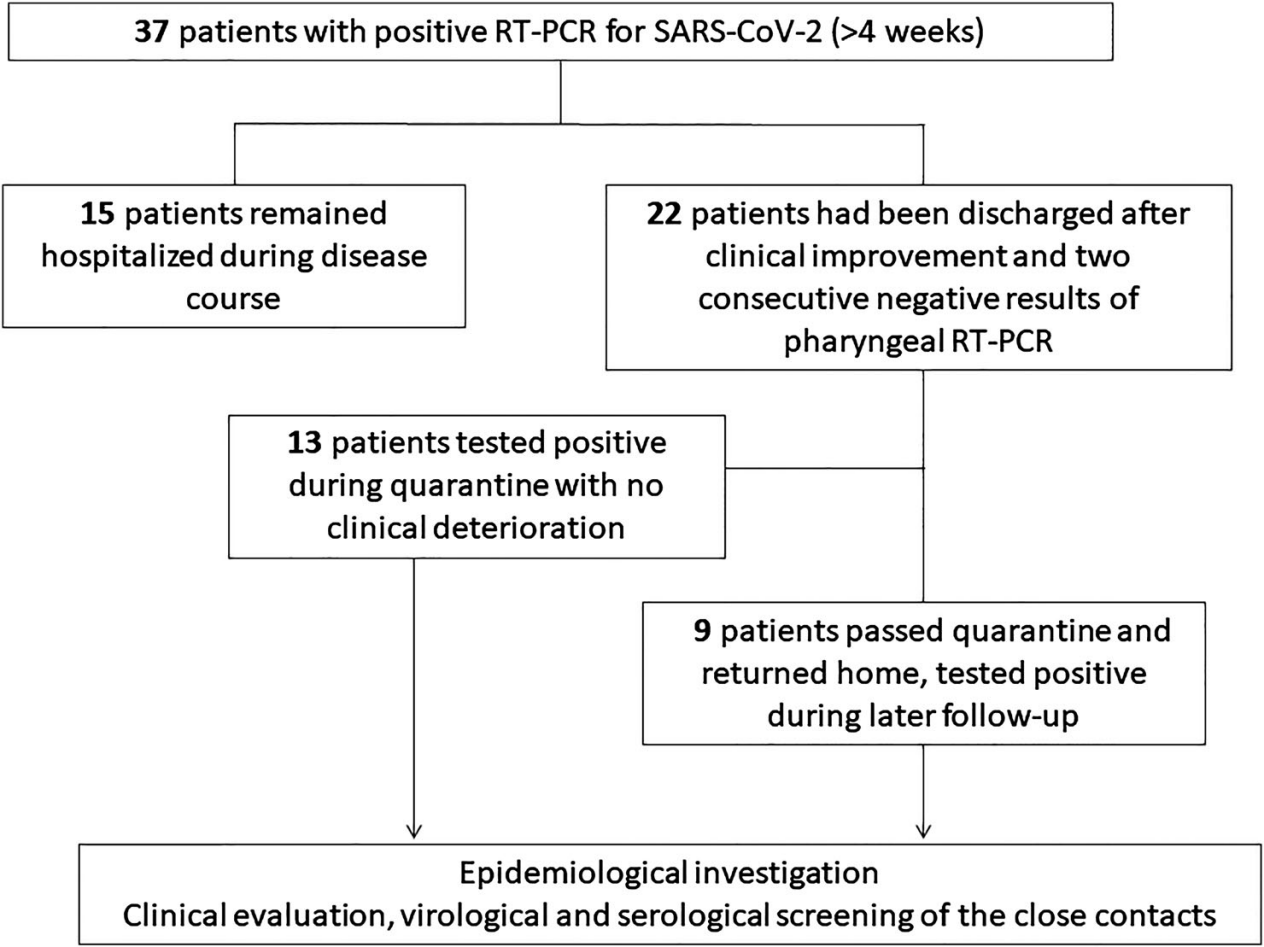
Flow chart of screening and follow-up of 37 COVID-19 patients with prolonged positive PCR results.

Among the 37 patients, 22 patients had been discharged following clinical and radiological improvement and two consecutive negative results of RT-PCR testing according to the discharge criteria at that time [7]. As a general procedure, discharged patients were quarantined for after-hospital observation for another 14 days before returning home. None of these 22 discharged patients showed recurrence or exaggeration of clinical symptoms including but not limited to fever, coughing, dyspnea, or gastrointestinal symptoms. However, they were tested positive for SARS-CoV-2 in later follow-ups, and were re-hospitalized for further observation. The other 15 patients remained hospitalized over the whole course, as they failed to show two consecutive negative PCR results required for discharge (Figure 1). For the convenience of description and discussion, these infected patients that have met the clinical standards of discharge, showing no apparent symptoms, or no exaggeration of residual symptoms or no onset of new symptoms, were considered clinical recovered or relieved patients with COVID-19 in the present study.

For all patients, the clinical, treatment and laboratory data were collected, and next generation sequencing (NGS) of respiratory samples was carried out at the last follow-up. SARS-CoV-2 immunoglobulin (Ig) M and IgG antibody testing was carried out for at least once during their hospital stay.

For those who had been discharged and re-admitted, their household contacts outside the hospital stay were carefully traced and recorded. Close contacts were defined as anyone who ever came within 2 m of a diagnosed patient without the use of effective personal protective equipment (including masks, gloves, goggles, and shields). For each close contact, a pharyngeal swab specimen was collected for RT-PCR testing regardless of symptoms, if the related patients developed positive virological testing results after discharge. At the same time, SARS-CoV-2 immunoglobulin (Ig) M and IgG antibody testing were also done for all close contacts.

This study complied with the declaration of Helsinki, and was approved by the Institutional Review Board of Jin-Yin tan Hospital. All subjects in this study gave their written informed consent.

### Real-time reverse transcription polymerase chain reaction (RT-PCR)

All the laboratory tests were carried out with lab certification of SAR-CoV-2 testing. Respiratory samples were collected and tested at first visit and during follow-up, including the nasopharyngeal, oropharyngeal and sputum samples. Molecular detection of SARS-CoV-2 was performed by real-time reverse-transcription PCR using commercial qualitative kits authorized by Chinese Centers for Disease Control and Prevention in the epidemic (Shanghai ZJ Bio-Tech Co., Ltd, Shanghai China). The lower limit of viral detection is 1×10^3^ copies/ml. The positive and negative coincidence were both 100% during clinical validation of the product among 252 samples of 199 individuals, according to manufacturer’s instructions.

### Next generation sequencing (NGS)

#### Construction of metagenomic sequencing libraries

For the preparation of metagenomic sequencing libraries, sample RNA was extracted from 200 μl sputum samples from patients using the nucleic acid extraction kit (Cat. MD005, MatriDx Biotech Corp. Hangzhou). Final RNA was eluted in 50 μl Elution buffer. Library preparation was performed by using 2019-nCoV Nucleic Acid Detection Kit (Cat. MD029, MatriDx Biotech Corp. Hangzhou) according to the manufacturer’s instruction. The reverse transcription master mix (6 μl cDNA first-strand synthesis buffer, 2 μl first-strand synthesis enzyme) was added into 12 μl RNA for each sample, and incubated at 25°C for 10 min followed by 50°C for 30 min and 75°C for 15 min. After cooling to 4°C, a second-strand synthesis master mix (4 μl cDNA second-strand synthesis buffer, 2 μl second-strand synthesis enzyme and 16 μl water) was added to each reaction, followed by a 16°C 30 min incubation. The resulting cDNA was cleaned up using 80 μl purification beads (Cat. MD012, MatriDx Biotech Corp. Hangzhou) and eluted in 37 μl water. 35 μl purified cDNA was then mixed with 10 μl fragmentation & end-repair buffer and 5 μl fragmentation & end-repair enzyme, followed by 37°C for 15 min and 75°C for 10 min. After cooling to 4°C, 30 μl adaptor and 5 μl DNA ligase was added to each reaction, followed by a 20°C for 15 min and 75°C for 5 min. The final library was purified by nucleic acid purification kit (Cat. MD012, MatriDx Biotech Corp. Hangzhou) using 42 μl purification beads. Libraries were pooled together according to the manufacture’s instruction and then sequenced on an Illumina NextSeq 500 system using a 75-cycle sequencing kit. A total of 10–20 million reads were obtained for each sample.

#### Bioinformatics analysis

Human genome was picked from Nucleotide Sequence Database (NT) and build kraken2 index. The software kraken2 was used to remove sequencing reads that mapped to the human genome in each sample, the parameter of confidence is equal to 0.5. Then the remained reads were aligned to the genomes of six kinds of human coronaviruses (SARS-CoV-2, SARS-CoV, MERS-CoV, NL63, OC43, 229E and HKU1), which were also extracted from NT database. After alignment, the aligned files in SAM format were parsed to count the number of sequencing reads for each of the species above while the multiply aligned reads were excluded.

### Anti-SARS-CoV-2-antibody detection

Detection of SARS-CoV-2 immunoglobulin M and immunoglobulin G was carried out by using a qualitative colloidal gold assay (Innovita (Tangshan) Biological Technology, Co., Ltd, Tangshan, China), following manufacturers’ instructions. The sensitivity of the assay was 87.3% (95%CI 80.4–92.0%), and the specificity was 100% (95%CI 94.20–100%) according to the instructions of the assay.

### Statistical analysis

Summary statistics were used to describe the study. Categorical variables were described as numbers (proportions) and continuous variables were described as medians and interquartile ranges (IQRs). The close household contacts of patients were calculated by person day spent with the patients. Data were analysed from 18 January to 18 April 2020.

## Results

### Demographic and clinical characteristics

A total of 37 patients were included in this study. Their median age was 62 years (IQR 50, 68), and 24 (64.9%) were men. Of these patients, 15 (40.5%) had hypertension, 8 (21.6%) had diabetes mellitus, 3 (8.1%) had chronic pulmonary diseases, 3 (8.1%) had chronic hepatis B virus infection, and 5 (13.5%) had a history of malignancy. Patients had common or severe type of COVID-19, and two of them received non-invasive ventilation during hospitalization [7]. Most patients were clinically stable despite persistence of viral RNA, and 29 (78.4%) denied any symptoms at last follow-up. Only one patient with relapsed epiglottis cancer continued to have fever, coughing and dyspnea due to recurrent pulmonary infections.

**Figure 2.**
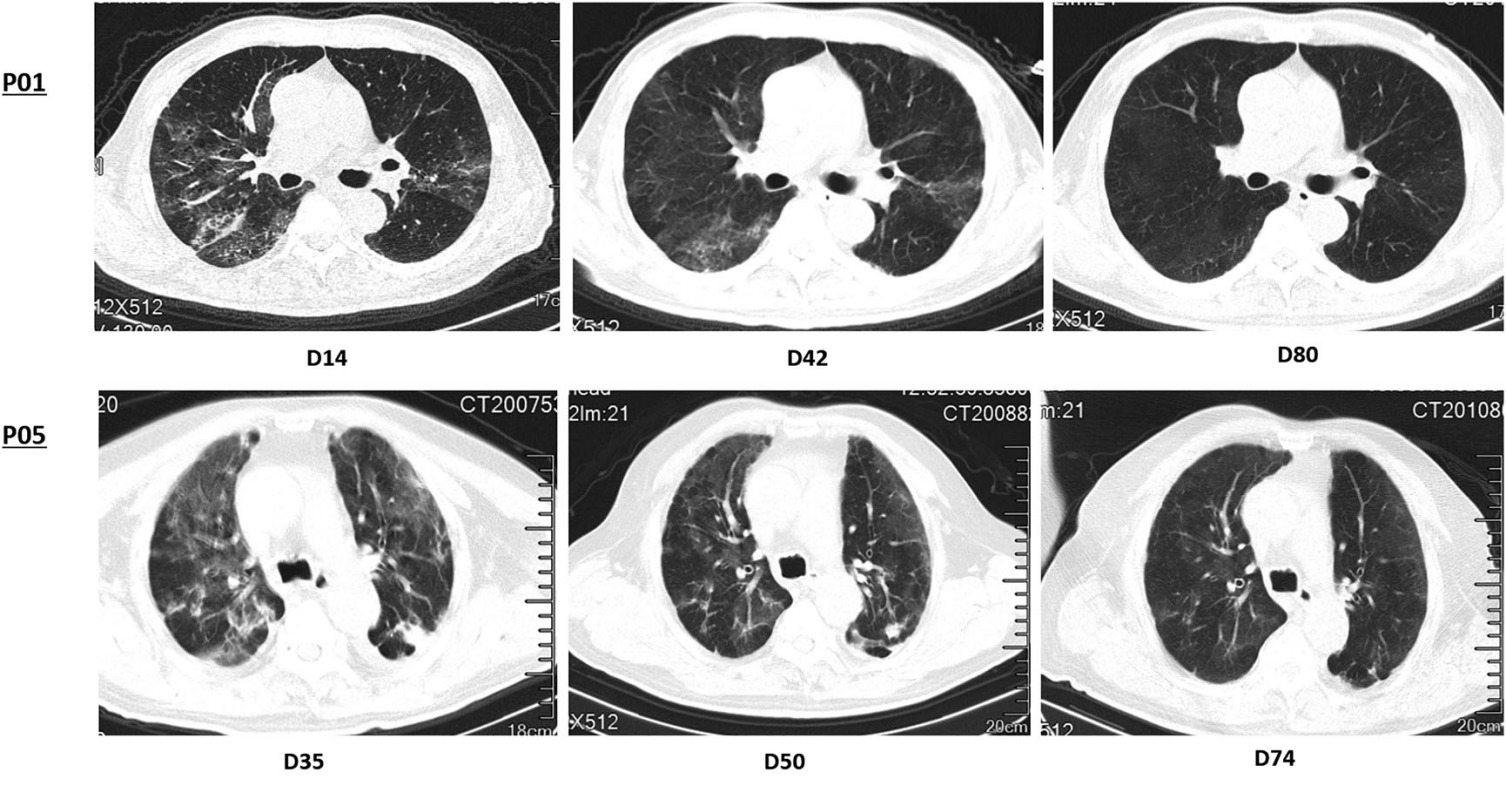
Series of chest CT presentation in patients with prolonged positive PCR results. Both patients did not present with any clinical symptoms. P01 showed typical changes of fully recovery, and P05 showed partial improvement at the time of evaluation.

Laboratory results at last follow-up were summarized in Table 1. Despite prolonged presence of viral RNA in the respiratory samples, the lymphocyte counts, including CD4 and CD8 T cells cell counts were generally within normal ranges. No obvious elevation was observed in measured inflammatory markers such as c-reactive protein, serum ferritin, interleukin (IL)-6. 32 patients had series of chest CT scans, with 2 (6.25%) recovered, 24 (75.0%) greatly improved, 5 (15.6%) stable, and one (3.13%) progressed in terms of interstitial fibrosis (Figure 2).

**Table 1.** Laboratory results of 37 COVID-19 patients with prolonged positive PCR results.

	Normal range	All patients (*n* = 37)
**White blood cell count, ×10^9^/L**	3.5–9.5	5.67 (4.81, 6.62)
**Neutrophil count, ×10^9^/L**	1.8–6.3	3.30 (2.67, 3.88)
**Lymphocyte count, ×10^9^/L**	1.1–3.2	1.75 (1.43, 2.34)
**CD4T cell count, ×10^9^/L**	345–2350	623 (533, 737)
**CD8T cell count, ×10^9^/L**	805–4459	428 (310, 567)
**Hemoglobin, g/L**	115–150	121 (106, 130)
**Platelet count, ×10^9^/L**	125–350	200 (167, 258)
**D-dimer, μg/mL, FEU**	0–1.5	0.32 (0.20, 0.65)
**Alanine aminotransferase, U/L**	7–40	17 (15, 28)
**Aspartate aminotransferase, U/L**	13–35	23 (19, 26)
**Total bilirubin, μmol/L**	0–21	9.6 (8.3, 11.1)
**eGFR, ml/min**	>90	113.26 (98.60, 133.22)
**Hypersensitive CRP, mg/dL**	0–6	0.32 (0.20, 0.65)
**Interleukin-6, pg/ml**	0–7	4.72 (4.01, 6.03)
**Serum ferritin, g/L**	4.63–204	144.8 (96.45, 188.73)

### Virological assessment

**Figure 3.**
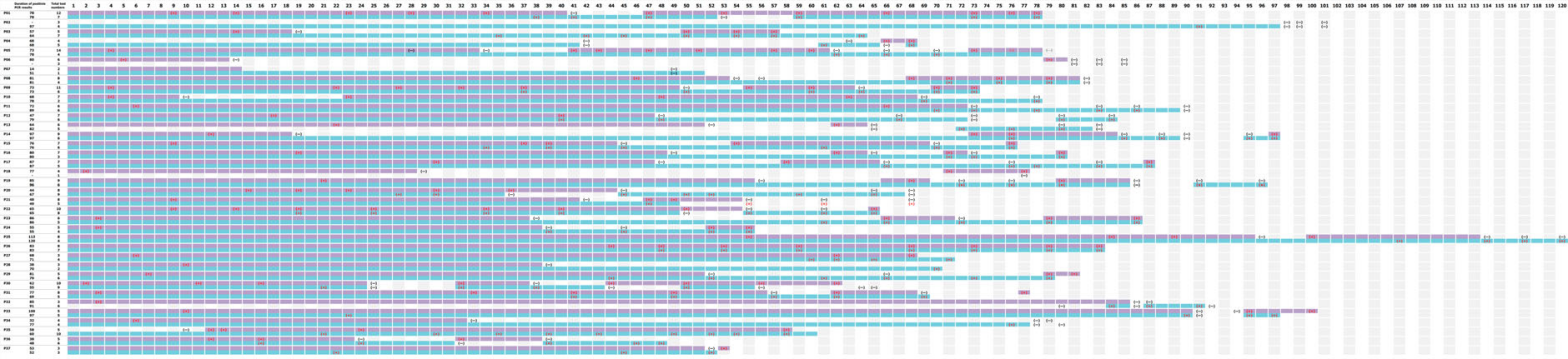
RT-PCR results of 37 COVID-19 patients with prolonged PCR positivity. The horizontal axis indicated the days after disease onset. PCR results of each patient (P01-37) were depicted by two lines of blocks. The first line of blocks indicted RT-PCR results from pharyngeal swabs, and the purple shade showed positive results. The second line of blocks indicted RT-PCR results from sputum samples, and the blue shade indicated positive results. The white zone in both lines indicated the time interval between testing negative for SARS-CoV-2 till the next positive testing. The total days of positivity and time of testing were also listed on the left of the figure.

All patients were closely followed for their virological status. A total of 431 PCR tests were carried out on pharyngeal swabs and sputum samples in this study, and patients were tested on a median of 8 time points each. The details of qualitative RT-PCR results from pharyngeal swabs or sputum samples for each patient were depicted in Figure 3. All 37 patients showed intermittent positive PCR results with respiratory samples. Moreover, the positivity of sputum samples was more consistent and stable compared with that of the pharyngeal swabs, as could be expected from the sampling procedure. The median time of PCR positivity up to last follow-up was 78 days (IQR 67.7, 84.5), and the longest duration of positive viral RNA was 120 days.

NGS for sputum samples from all 37 patients was done at a median of 80 days from disease onset. Among them, 24 patients got zero SARS-CoV-2-specific reads. The number of reads corresponding to SARS-CoV-2 in the other 13 patients ranged from 1 to 92, with genomic coverage from 0.2475% to 20.1752% (Table 2 and Supplementary Table S1), in contrast to the high volume and near-full coverage of viral genome at the beginning of infection (data not shown).

**Table 2.** Next-generation sequencing (NGS) of the sputum in 13 patients*.

Patient	Days from onset to sputum collection	Total reads	SARS-CoV-2-specific reads	Genomic coverage (%)
**P04**	67	15362103	5	1.2541
**P05**	82	15493848	7	1.749
**P06**	84	18180220	4	1.0032
**P16**	79	20450201	7	1.3544
**P17**	87	16818744	1	0.2542
**P20**	68	12254239	1	0.2475
**P22**	67	19233766	4	1.0032
**P25**	120	11264897	1	0.2508
**P26**	85	12998557	18	4.1735
**P27**	68	17147763	2	0.5016
**P29**	81	17410324	1	0.2508
**P31**	77	17193425	92	20.1752
**P36**	48	20418210	1	0.2508

*NGS of the other 24 patients had the results of 0 SARS-CoV-2-specific reads. Results of all patients were shown in Supplementary Table S1.

In addition, all patients have been tested at least once after one month of infection for immunoglobin G (IgG) and IgM antibodies against SARS-CoV-2 (Supplementary Table S2). Among them, 17 patients were tested twice during hospitalization. All patients were positive for anti-SARS-CoV-2 IgG at all testing points, ranging from 26 days to 101 days post symptom onset. In five patients (P3, P9, P11, P30, P36), sequential loss of anti-SARS-CoV-2 IgM antibody were observed mostly within the third month of infection, whose IgG antibody remained consistently positive in accordance to a post-infection status.

### Contact tracing

Twenty two of the 37 patients had been discharged according to the criteria consisting of clinical relief and two consecutive negative RT-PCR results. The median time of discharge in these patients was 44 days (IQR 22.3, 50 days) from disease onset. Of them, 13 patients were found recurrence of PCR positivity for at least once during the 14-day quarantine for after-hospital observation with no reported close contacts. The other 9 patients had finished 14-day quarantine and returned home.

They had lived with their family members without personal protections for a total of 258 person-days. No close contacts showed any signs of symptomatic infection. For all 13 close contacts, RT-PCR of pharyngeal swabs and IgM/IgG antibodies against SARS-CoV-2 were done after re-admission of related patients. None of these tests were positive.

## Discussion

The COVID-19 pandemic is still progressing with growing numbers of confirmed patients globally. Having a different pattern of viral dynamics from prior coronaviruses, SARS-CoV-2 has been reported to exhibit prolonged period of detectable viral nucleic acid in certain COVID-19 patients, despite greatly improved clinical symptoms. Clinical features of patients with persistent or intermittent positive respiratory PCR testing have been reported in a few studies, however, none of them discussed the epidemiological significance of these cases, and adequate disease control approach towards this specific population remains unclear. Our long-term observations showed that prolonged presence of SARS-CoV-2 RNA was not rare in recovered patients with COVID-19. Although some patients showed intermittent positivity, it was most probable due to methods of sampling at different settings [8]. In our study of patients with resolved clinical symptoms, prolonged viral RNA in upper respiratory tract did not result in secondary infection despite close and unprotective household contact. Therefore, disease prevention measures in these individuals should be re-considered.

The basic reproductive number (the number of cases one case generates on average over the course of its infectious period, i.e. R0) of SARS-CoV-2 has been estimated to be at 2.2 to 5.7, much higher than that of other fatal coronaviruses [9–11]. Therefore, the exact time of viral spreading in affected patients and relevant infection prevention measures are extremely important in controlling the epidemic. Recent studies on viral dynamics have demonstrated that viral shedding from the respiratory tract peaked in the first week of SARS-CoV-2 infection, and subsided after three weeks in most cases [1–3]. However, more and more patient series with prolonged respiratory viral RNA were observed and reported with pandemic progression [4,6,12,13]. The reported longest period of positive viral PCR testing was up to 45 days post infection. Our study also confirmed that prolonged viral RNA was not rare in COVID-19 patients, and could last for quite a period. In all these patients, symptoms were stable or continued to improve. All reports agreed that “recurrence” of viral RNA was unlikely to be due to reinfection, and few such patients were associated with recurrence of clinical symptoms at the same time. Some also retrospectively defined several risk factors associated with prolonged viral positivity [6,13]. However, in most of these studies, PCR testing was used as a marker to indicate the existence of virus, and patients with positive viral RNA was considered infectious though they have been infected for months without further clinical symptoms.

While presence of viral genetic element constitutes the basic condition of effective transmission, traces of virus detected by RT-PCR or NGS could not be readily translated into intact and transmissible virus. In fact, infectiousness of affected patients may decline significantly after 8 days of symptom onset, as live virus could no longer be cultured from then on [1]. Our results of the PCR testing during relatively long follow-up suggested that results of this assay should be interpreted in a dynamic manner. However, with prolongation of disease course, PCR testing may not take as important a role as it was shortly after infection. The very limited reads and coverage of NGS results in our patients, compared with abundant reads and near-full genome coverage in newly infected patients, partially indicated greatly reduced integrated virus in these patients. Unfortunately, no current virological measurement including PCR testing, NGS analysis or even viral culture could truly reflect infectiousness. Therefore, although we provided the viral evaluation results in these patients, critical evidence came from that no secondary infection was resulted from those patients who had substantial household contacts with their family members. This strongly advocates the fact that despite prolonged viral RNA in respiratory samples of these patients, they would not cause effective transmission. This was also supported by the fact that although positive respiratory viral testing was reported in recovered patients from time to time in Wuhan, related secondary cases haven’t been confirmed following lifting Wuhan’s lockdown.

Our result is also consistent with recent model for the infectiousness of SARS-CoV-2, which showed increased infectiousness of COVID-19 around symptom onset and a quick decline afterwards [14]. From our clinical observations of these patients with prolonged viral RNA presence, we speculated that infectiousness of such patients should be evaluated considering the disease course and clinical status. Those patients who have been infected for more than four weeks with clinical recovery, are probably not a source of infection regardless of the presence of viral RNA.

Our study has its limitation that a relatively small number of patients were included, which makes it difficult to associate any demographic features with increased risk of prolonged viral RNA in COVID-19 patients. Nevertheless, no apparent immunocompromise was identified in most patients. In addition, tests that could accurately define or rule out the capacity of viral transmission is still lacking. Under this circumstance, the epidemiological evidence may be the best clinical demonstration of non-infectiousness.

In summary, our study showed that prolonged presence of viral RNA could be detected in certain SARS-CoV-2 infected patients. However, this does not necessarily mean prolonged period of active infectiousness, which should not be solely evaluated by PCR testing. In contrast, those who have clinically recovered and whose disease course has exceeded four weeks were associated with very limited infectiousness. Control measures for these patients may need reconsidered, and more evaluation of virological status in such patients is warranted.

## Supplementary Material

Supplementary_Appendix.docx
