# Supplementary material for: Prolonged presence of viral nucleic acid in clinically recovered COVID-19 patients was not associated with effective infectiousness
Source: Emerg Microbes Infect. 2020 Oct 27;9(1):2315–21. doi: 10.1080/22221751.2020.1827983 (PMC7594837; doi:10.1080/22221751.2020.1827983)
Supplement: Supplementary_Appendix.docx [file TEMI_A_1827983_SM6330.docx]

**Table S1 Next-generation sequencing (NGS) of the sputum in all 37 patients**

| Patients | Days from onset to  sputum collection | Total reads | Genomic coverage (%) |
| --- | --- | --- | --- |
| P01 | 79 | 15257089 | 0 |
| P02 | 100 | 16905302 | 0 |
| P03 | 63 | 12686071 | 0 |
| P04 | 67 | 15362103 | 1.2541 |
| P05 | 82 | 15493848 | 1.749 |
| P06 | 84 | 18180220 | 1.0032 |
| P07 | 48 | 17860154 | 0 |
| P08 | 81 | 17750101 | 0 |
| P09 | 72 | 13251688 | 0 |
| P10 | 84 | 11959957 | 0 |
| P11 | 89 | 13652160 | 0 |
| P12 | 84 | 19746397 | 0 |
| P13 | 82 | 11545164 | 0 |
| P14 | 97 | 11098946 | 0 |
| P15 | 76 | 17229648 | 0 |
| P16 | 79 | 20450201 | 1.3544 |
| P17 | 87 | 16818744 | 0.2542 |
| P18 | 75 | 15717436 | 0 |
| P19 | 96 | 9165603 | 0 |
| P20 | 68 | 12254239 | 0.2475 |
| P21 | 80 | 13406247 | 0 |
| P22 | 67 | 19233766 | 1.0032 |
| P23 | 86 | 14534351 | 0 |
| P24 | 55 | 11523244 | 0 |
| P25 | 120 | 11264897 | 0.2508 |
| P26 | 85 | 12998557 | 4.1735 |
| P27 | 68 | 17147763 | 0.5016 |
| P28 | 74 | 16940640 | 0 |
| P29 | 81 | 17410324 | 0.2508 |
| P30 | 62 | 12769784 | 0 |
| P31 | 77 | 17193425 | 20.1752 |
| P32 | 93 | 16155196 | 0 |
| P33 | 97 | 17279582 | 0 |
| P34 | 80 | 28851927 | 0 |
| P35 | 60 | 16648786 | 0 |
| P36 | 48 | 20418210 | 0.2508 |
| P37 | 50 | 15971690 | 0 |

**Table S2. Anti-SARS-CoV-2 antibody profiles of patients with prolonged viral positivity**

| Patients | Days from disease onset | Anti-SARS-CoV-2 IgM | Anti-SARS-CoV-2 IgG |
| --- | --- | --- | --- |
| P1 | 43 | + | + |
| P2 | 99 | + | + |
|  | 101 | + | + |
| P3 | 44 | + | + |
|  | 64 | - | + |
| P4 | 25 | + | + |
|  | 63 | + | + |
| P5 | 44 | + | + |
|  | 63 | + | + |
| P6 | 81 | + | + |
| P7 | 49 | - | + |
| P8 | 71 | + | + |
| P9 | 49 | + | + |
|  | 73 | - | + |
| P10 | 71 | + | + |
|  | 85 | + | + |
| P11 | 70 | + | + |
|  | 90 | - | + |
| P12 | 79 | + | + |
| P13 | 68 | + | + |
| P14 | 73 | - | + |
|  | 98 | - | + |
| P15 | 33 | - | + |
|  | 78 | - | + |
| P16 | 65 | - | + |
| P17 | 64 | + | + |
| P18 | 71 | - | + |
| P19 | 68 | - | + |
| P20 | 43 | - | + |
| P21 | 55 | - | + |
| P22 | 30 | + | + |
|  | 51 | + | + |
| P23 | 64 | + | + |
|  | 89 | + | + |
| P24 | 39 | + | + |
|  | 58 | + | + |
| P25 | 83 | + | + |
| P26 | 48 | + | + |
|  | 88 | + | + |
| P27 | 62 | - | + |
| P28 | 76 | - | + |
| P29 | 44 | - | + |
| P30 | 33 | + | + |
|  | 63 | - | + |
| P31 | 43 | - | + |
|  | 78 | - | + |
| P32 | 86 | - | + |
| P33 | 91 | - | + |
| P34 | 79 | + | + |
| P35 | 26 | + | + |
|  | 59 | + | + |
| P36 | 26 | + | + |
|  | 49 | - | + |
| P37 | 52 | - | + |

* Five patients (P3, P9, P11, P30, P36) showed sequential loss of anti-SARS-CoV-2 IgM antibody, mostly within the third month of infection, whose IgG antibody remained consistently positive.
